# A Preliminary Study: Central Vestibular Sensitivity Affects Motion Sickness Susceptibility through the Efficacy of the Velocity Storage Mechanism

**DOI:** 10.4081/audiores.2020.245

**Published:** 2020-08-19

**Authors:** Kenneth Chua Wei De, Kek Tze Ling

**Affiliations:** 1 Department of Allied Health, Audiology, Otorhinolaryngology, Changi General Hospital, 2 Simei St 3, Changi General Hospital, Singapore 529889, Singapore; 2 Department of Otolaryngology, Audiology, National University Hospital, National University Health System, 5 Lower Kent Ridge Rd, Singapore 119074, Singapore

**Keywords:** motion sickness susceptibility, velocity storage mechanism, vestibular, time constant, slow-phase eye velocity, perceived rotational duration

## Abstract

*Background/Objective*: Slow-Phase Eye Velocity Time constant (SPEV TC) and Perceived Rotational Duration (PRD) are measurable objective outcomes of rotational chair step-velocity test. These two variables are dependent on the efficacy of the central velocity storage. If sensory conflict from the step-velocity of the rotational chair elicits motion sickness, the SPEV TC and PRD in individuals with varying susceptibility to motion sickness should be affected. We determined if Central Vestibular Sensitivity (CVS) characteristics differ among individuals with a range of Motion Sickness Susceptibility (MSS). *Methods*: Participants were allocated to two groups based on MSS (low and high) as identified on the short version of the Motion Sick Susceptibility Questionnaire (MSSQ-S). We evaluated the specific relationship between MSS and the characteristics of CVS through the SPEV TC and PRD from the step-velocity test. *Results*: Results showed significant differences in the PRD between these two groups. 180°/s Per-rotatory PRD is most significantly different (*p* = 0.005) followed by 50°/s post-rotatory PRD (CCW, *p* = 0.007; CW, *p* = 0.021) and log of 180°/s post-rotatory PRD (*p* = 0.042). Multiple regression analysis indicated that CCW post-rotatory PRD at 50°/s was a strong predictor of MSS. *Conclusions*: High MSS individuals were observed with elevated PRD in general, indirectly suggesting greater velocity storage efficiency, hence, greater CVS; CVS is therefore positively correlated with MSS. PRD could be a reliable clinical indicator of motion sick susceptibility and may help with the selection of personnel working in motion sick environments and with the verification of motion sickness therapeutic interventions.

## 1. Introduction

It has long been suggested that Motion sickness Susceptible (MSS) individuals are inclined to possess vestibular systems that are more sensitive as compared to individuals less prone to motion sickness. By inquiring into the source of variability among individuals in MSS, mechanisms that are involved in its incidence can be identified, assisting the development of prediction tests of MSS [1]. This will not only aid in the selection of personnel required to work in motion environments, but also help in improving the quality of life for individuals prone to motion sickness. The sensitivity of the Central Vestibular System (CVS) plays a critical role in the production of motion sickness [2]. While the etiology of motion sickness remains elusive, the theory of sensory conflict is widely accepted as the most common cause for motion sickness [3]. Sensory conflict results from the mismatched integration of multiple sensory inputs (proprioceptive, visual and vestibular) at the level of neural integrators. Among the many neural integrators in the brain, sensory conflict is known to result from one integrator, which is called the Velocity Storage Mechanism (VSM), located at the central vestibular system. While the VSM functions to integrate sensory information from multiple inputs, its primary function is to prolong the Slow-Phase Eye Velocity Time Constant (SPEV TC) and Perceived Rotational Duration (PRD) in relation to motion, to compensate for the vestibular system’s lack of sensitivity in detecting low frequency movements [4].

As such, SPEV TC and PRD are responses that can be measured as an indirect evaluation of the VSM’s efficiency. Although the exact relationship between SPEV TC, PRD, and MSS has yet to be characterized, it is postulated that the efficiency of the VSM in modulating SPEV TC and PRD determines the propensity for sensory conflicts to occur. Hence, this study aims to find out if CVS (determined by VSM efficiency) would indirectly correlate to propensity for sensory conflicts, in turn affecting MSS. As SPEV TC and PRD are indirect measurements of CVS, we hypothesize that SPEV TC (reflex) and PRD (perceptual) are elevated in MSS individuals because of increased CVS. This elevation should demonstrate a positive correlation between the degree of MSS and the perceptual reflex durations. The role of the CVS in the production of motion sickness is understood from several habituation studies that have assessed the correlation between SPEV TC and MSS after exposure to habituation training [5]. The eye movement duration decreased alongside MSS; however, little is known about perceptual responses. It is unknown if people who are highly susceptible to motion sickness will have a longer reflexive eye movement and/or perceptual responses, thus, the exact nature of the relationship between motion sickness and the perceptual-reflex response is still unclear.

Prior studies have looked at the perceptual-reflex responses separately, without a direct recording and comparison of these two responses. Past reports were also conflicting, showing significant changes to the reflexive eye movement duration in motion sickness subjects [1], and no changes in a similar study two years later [6].Therefore, to support the hypothesis, we directly recorded the perceptual-reflex responses and compared them between the two groups of high and low MSS.

## 2. Materials and Methods

This is a quantitative descriptive correlation study to assess the effects of Central Vestibular Sensitivity (CVS) on Motion sickness Susceptibility (MSS) in healthy individuals, through the function of Velocity Storage Mechanism (VSM). The study was approved by the Bioethics Advisory Committee of the National Healthcare Group (NHG) domain specific review board, in compliance with requirements based on the declaration of Helsinki and ethical principles of the Belmont Report. Participants were recruited from a university and hospital wide exercise at the National University Health Services (NUHS) from June 2014 to February 2015.

All participants were first screened with audiometry (to ensure no asymmetrical hearing loss), tympanometry, video head impulse (vHIT), cervical- vestibular evoked myogenic potential (c-VEMP), videonystagmography (VNG) that includes oculomotor tasks (smooth pursuit, saccades and bi-directional optokinetic), positional and post-headshake tests, Dix-Hallpike, spontaneous and gaze evoked nystagmus tests for any peripheral or central vestibular deficits. The c-VEMP and vHIT ensured the integrity of both the superior and inferior vestibular nerves were intact. Although the caloric test was not carried out, such an aphysiological test of vestibular function, (0.001 Hz to 0.003 Hz) had little clinical significance for a compensated peripheral vestibular dysfunction. The step-velocity test in the main experimental set-up, taken together with the vHIT, VNG and neurotologic history is sufficient to rule out any significant uncompensated peripheral vestibular problems in the physiological ranges of movement. The ocular-vestibular evoked myogenic potential (o-VEMP) was not carried out, as there was a lack of expertise in this procedure and we also had no local normative data to take reference from. In addition, it was acknowledged that although possible, an isolated end-organ utricular dysfunction is a rare occurrence. The participants were then administered the Motion sickness Susceptibility Questionnaire-Short (MSSQ-S). This questionnaire is designed to find out how susceptible to motion sickness one is, and what kind of motion are most effective at causing sickness [7].They were then subjected to an earth-vertical axis rotation (EVAR) on a rotatory chair, in the main experimental step-velocity test.

### 2.1. Main Experimental Protocol: Step- Velocity

Two specific outcomes measures were obtained from this test; the slow-phase eye velocity time constant (SPEV TC) as well as the perceptual rotational duration (PRD). SPEV TC and PRD directly quantifies how efficient the velocity storage mechanism (VSM) is at storing peripheral vestibular signals and hence, indirectly qualifies the sensitivity of central vestibular system (CVS).

Participants were seated on a motorized friction free rotating chair (Interacoustic, Nydiag 200, Middelfart, Denmark) fitted with head, foot and arm rests. All participants were strapped in with a safety belt and their legs were held firmly with a Velcro strap. They were fitted with the horizontal binocular VN415 (Interacoustic VNG) to record their eye movements. Participants were instructed to make saccades and track the target dot on the screen to calibrate their eye movements. Two stimulus velocities were chosen for the rotation. The main velocity of interest was the high-speed rotation at 180°/s because it stimulated real-life conditions of fast movement [8]. For consistency and test reliability, the high-speed rotation was conducted four times, twice in each clockwise (CW) and counter clockwise direction (CCW). As a within experiment control to determine if the speed of rotation influences the outcome measures, a slower stimulus velocity of 50°/s was also used for the remaining two rotations.

A total of six rotations lasting sixty seconds each were conducted. Both per and post rotatory eye movements were recorded. Participants were all given similar instructions, to keep their eyes open at all time, with head slightly tilted down and secured by a head strap to put the horizontal semi-circular canal in the right plane for angular rotation. Eye movements were recorded in the dark with binoculars covered as visual fixation will affect the results.

To evaluate the perceptual rotational duration (PRD), participants were asked to click on an infrared connected computer mouse, strapped on the armrest four times with each rotation at different time intervals. The first click is delivered by the participant when the chair starts rotating (per-rotatory). This starts the timer, and the second click is delivered the moment the participants feel that the spinning sensation has disappeared during the rotation. Once the chair stops spinning after 60 s, the participants deliver the third click, to start timing the duration of spinning in the opposite direction (post-rotatory). The final click is delivered when the spinning sensation completely disappears after the chair has come to a stop. Each click of the computer mouse switched on or off a fixation light emitter and measured the start or stop of the perceptual spinning for both per and post rotation in real time. The PRD will then be reflected as a green light bar, synchronized in time with the SPEV TC measurements. The nystagmus recordings and chair motion were stored at 250 Hz sampling rate. Signals were calibrated and differentiated to remove the fast phase of eye movements and averaged from the offset of the chair velocity to generate the slow-phase eye velocity (SPEV) curve. To prevent the suppression of nystagmus during the SPEV recording due to fixation light, the light emitter was covered with opaque stickers. Participants were also given a mental task during the tests to maintain mental alertness to reduce the influence of anxiety and attention on the time constants [9].All data for per and post-rotatory for SPEV TC and PRD were recorded down in an excel sheet for statistical analysis.

### 2.2. Data Analysis

Where the distribution of the SPEV TC and PRD independent variables were normally distributed, parametric paired sample and independent sample T-tests were conducted. However, where the variables were severely skewed (*p* < 0.01), non-parametric tests such as the Wilcoxon signed rank test and Mann-Whitney U test were used instead. Depending on whether there are significant differences in the SPEV TC and PRD between ears, the outcome measures are either averaged or looked at separately with ear-specific comparison between the subject and control group. Independent *t*-tests were conducted to determine if there were significant difference in the SPEV TC and PRD between control and subject group at *p* value of 0.05 with 95% confidence interval.

The significant correlation between degree of MSS and various independent variables were determined through stepwise forward regression method, using the Akaike information criterion (AIC) values to evaluate the quality of the independent variables relative to each other.

### 2.3. Recruitment

Fifty volunteers were recruited and there were 15 males and 35 females. The median age of the participants was 26.0 years (SD = 6.67) and there was no significant difference in the median age between gender. A basic history was first taken by a trained physician in the Ear-Nose Throat (ENT) department, with bedside tests of cerebellar, cranial nerves and focal neurological deficits to exclude central pathologies, mal-de-debarquement syndrome, migraine, any neurotologic, psychiatric, significant cognitive impairment (Abbreviated Mental Test < 7) or non-corrected visual deficits. Based on literature review, participants with severe visual impairment were excluded as low vision can result in a number of symptoms that will overlay with motion sickness such as dizziness, nausea and headaches. Participants on long-term anxiety or depression medications or who have abnormal vestibular screening test results were also excluded. Participants filled up a bio-data information sheet to include information such as gender, age, severity rating of their motion sickness from 0–10; Likert scale [10] and number of motion sickness symptoms reported (MS ratings group). Participants were blinded and were assigned to either the control group (< 80th percentile, *n* = 27) or highly motion sickness susceptible group (>/= 80th percentile, *n* = 20) based on their MSSQ-S scores. Three participants were excluded due to a significant history of either chronic migraine, Mal-De-Debarquement syndrome or psychiatric condition. As such, only forty-seven participants’ data were recorded. We present the results for slow (50°/s) and fast speed rotation (180°/s) separately. Final values for independent *t*-tests comparison between subject and control groups for slow speed rotation (50°/s) were, CW and CCW per-rotatory SPEV TC, averaged (CW + CCW) post-rotatory SPEV TC, averaged (CW + CCW) per-rotatory PRD, CW and CCW post-rotatory PRD.

## 3. Results

### 3.1. 50°/s

After excluding results with poor gains and/or significant asymmetry between ears, there were (*N* = 14) subjects and (*N* = 15) controls with complete data for slow-speed analysis. Data included for analysis at this point were not significantly asymmetrical between ears, as indicated by the step-velocity test results and paired-sample T-tests conducted previously.

#### 3.1.1. SPEV TC

The results showed moderate evidence of significant differences for the CW per-rotatory SPEV TC between the control and subject group, with a mean difference of 3.10 s (2 dp) [Control- MSS], (*p* = 0.012, 95% CI; 0.73, 5.46). No significant differences were observed for the other two variables: CCW per-rotatory (*p* = 0.516) and averaged post-rotatory SPEV TC (*p* = 0.990) between the control and subject group.

#### 3.1.2. PRD

There were statistically significant differences for the CW post-rotatory PRD, (*p* = 0.021, 95% CI; −18.1, −1.59; 2 dp) with a mean difference of −9.84 s (2 dp) between the control and MSS group. Statistically significant differences were also observed for the CCW post-rotatory PRD, (*p* = 0.007, 95% CI; −22.56, −3.96; 2 dp) with a mean difference of −13.26 s (2 dp) between control and subject group. No statistical differences were observed for the averaged (CW + CCW) per-rotatory PRD values between the control and MSS group (*p* = 0.502). A summary of all SPEV TC and PRD findings for slow-speed rotation (50°/s) is in Table 1.

### 3.2. 180°/s

For fast speed rotations (180°/s); first recorded CW and CCW per-rotatory, second recorded averaged per-rotatory (CW + CCW) SPEV TC, total averaged (1st and 2nd recorded CW + CCW) post-rotatory SPEV TC and the total averaged per and post rotatory PRD respectively were selected.

#### 3.2.1. SPEV TC

No statistically significant differences were observed for all SPEV TC variables. All *p* values, were greater than at the significance level of *p* = 0.05, suggesting strong evidence against the null hypothesis, in favour of the alternate that there are no differences in the SPEV TC between the control and subject group for high-speed rotations.

#### 3.2.2. PRD

The results displayed strong evidence against the null hypothesis for Total averaged (1st and 2nd recorded) per-rotatory PRD (*p* = 0.005, 95% CI; −17.86, −3.64; 2 dp) with a mean difference of −10.75 s (2 dp) between the control and MSS group. There was, however, less evidence of statistical difference observed for Total averaged (1st and 2nd recorded) post-rotatory PRD. Evidence of differences, against the null hypothesis (*p* = 0.042, 95% CI; −21.87, −0.45; 2 dp), was only moderate between the control and subject group, with a mean difference of −11.16 (2 dp) seconds. A summary of the outcome measures for fast-speed rotation is in Table 2.

The final few independent variables (IV) selected for the linear regression model after bivariate correlation analysis and using AIC values were for 50°/s: CW per-rotatory SPEV TC, CCW post-rotatory PRD, Gender and averaged per-rotatory PRD. For 180°/s; motion sickness rating, gender, total averaged post-rotatory SEPV TC and log of total averaged post-rotatory PRD.

### 3.3. Linear Regression for 50°/s

The adjusted R2 analysis suggests that the independent variables in the model, such as CCW Post-rotatory PRD, CW Per-rotatory SPEV, Gender and Averaged Per-rotatory PRD explained for 34.3% (0.343) of the total variability in the outcome dependent variable (MSSQ percentile).

A highly significant *p* value (*p* << 0.05) strongly rejects the null hypothesis in favour of the alternative. There is evidence that the independent variables stated, had predictive powers over the outcome of interest (MSSQ percentile). Using the enter method, the results showed that these independent variables explained for a significant amount of the variance in the MSSQ percentile [F (4.24) = 4.657, *p* << 0.05, R2 = 0.437, R2Adjusted = 0.343]. The analysis showed that both CW Per-rotatory SPEV TC (*p* < 0.05) and CCW Post-rotatory PRD (*p* << 0.05) did significantly predict the value of MSSQ percentile.

#### 3.3.1. CW Per-rotatory SPEV TC

Moderate evidence of negative correlation (*p* = 0.039, 95% CI; −6.87, −0.19; 2 dp), mean difference: −3.5 s (1 dp).

#### 3.3.2. CCW Post-rotatory PRD

Strong evidence of positive correlation (*p* = 0.002, 95% CI; 0.57, 2.25; 2 dp), mean difference: 1.41 s (1 dp). The rest of the variables were not strong predictors and is shown below in Table 3.

### 3.4. Linear Regression for 180°/s

The Independent Variables (IVs) such as Log of total averaged post-rotatory PRD, Gender, total averaged post-rotatory SPEV TC and Motion sickness ratings group explained for 57.2% (0.572) of the total variability in the dependent variable (DV). The *p* value was highly significant (*p* <<< 0.05), thus showing there was strong evidence that the IVs had predictive powers over the DV. Using the enter method, it was suggested that these independent variables explained for a significant amount of the variance in the MSSQ percentile [F (4.32) = 13.037, *p* << 0.05, R2 = 0.620, R2Adjusted = 0.572].

#### Significant Predictors

MS Ratings Group; very strong evidence of positive correlation (*p* = 0.000, 95% CI; 32.48, 65.45), mean difference of 49.0 (1 dp). The rest of the variables were not strong predictors; Table 4.

## 4. Discussion

### 4.1. SPEV TC

We did not expect any significant results from the slow speed rotation of 50°/s, as they are meant to be a within experimental control. The purpose of the slow-speed rotation was to establish a baseline of reference for the fast speed rotation at 180°/s. If there were significant differences in the SPEV TC between the control and subject group at 50°/s, differences observed at 180°/s may have been because of other factors, such as the otolith’s involvement [6], abnormal posture [11] and anxiety [12] affecting the time constants. Although migraine [13] can be an influencing factor on time constants, it is irrelevant in this study as patients with migraine were excluded.

However, CW per-rotatory SPEV TC were significantly different and negatively correlated at a moderate level of statistical evidence. This observation was not consistent with the hypothesis that highly motion sickness susceptible subjects have elevated SPEV TCs. On the contrary, the result suggested that participants in the subject group had an average of 3.1 s shorter SPEV TCs, compared to the control group (Independent T test). Since participants were always subjected to the CW per-rotatory experience at the slow-speed first, the initial rotatory experienced might have induced anxiety. More participants in the motion sickness subject group also reported anxiety and fear before the experiment was conducted and may have supressed their nystagmus leading to a shorter SPEV TC. No such differences were observed for the CCW per-rotation. Moreover, the dynamics of the fluid movement in the semi-circular canals between ears are believed to be similar, this inconsistency observed for CW per-rotation SPEV TC could be due to effects of anxiety and novelty of being exposed to the initial rotatory experience.

We looked at fast speed rotation of 180°/s, which more closely resembles real life motion of daily living [1]. Slow speed rotation of 50°/s though sufficient to provoke the vestibular system, may not be fast enough to elicit symptoms of motion sickness, within a short 60 s rotation timeframe. Consistent with prior studies, there are generally no significant differences in SPEV TCs for slow speed rotations. For 180°/s, all per and post rotations for CW and CCW directions had no significant differences in SPEV TC between control and subject group. SPEV TC was hence noted to be a poor predictor of motion sickness susceptibility, regardless of speed of rotation after regression analyses were conducted. Prior studies conducted by [1,6], with the same experimental set-up at the rotational speed of 105°/s showed conflicting results. The present results thus supported the fact that while the vestibular ocular system (eye movement) displayed an overall predisposition to MSS, there is no predisposition specifically to any one type of stimulation [6].It is possible that the effects of speed did not matter and instead, the effects of different head tilt angles in the Off-Vertical Axis Rotation (OVAR) may have contributed to his different observations.

### 4.2. PRD

As PRD shares a similar velocity storage pathway as SPEV [14], no difference in PRD for slow-speed rotation were expected to be like SPEV TC between the control and subject group (*p* > 0.05). The only difference found was for 50°/s CW post-rotatory PRD that could have been due to anxiety since this was always the first post-rotatory experience for the slow-speed rotation, like the first CW per-rotatory experience for 50°/s for which, significant differences were observed for SPEV TC.

However, unlike SPEV TC, significant differences for post-rotatory CW and CCW PRD were observed at fast-speed rotations. This is likely due to the velocity storage playing a greater role in the post-rotatory experience as compared to per-rotation [2]. Post-rotatory CCW PRD even at a slow speed rotation, was a significant predictor (*p* = 0.007) of motion sickness susceptibility, consistent with the hypothesis that there is a significant correlation between degrees of motion sickness and PRD. For fast speed rotations, both per and post-rotatory PRD were significant predictors of motion sickness susceptibility.

However, because significant differences were found at 50°/s for post-rotatory PRD, the differences in log of post-rotatory PRD between the control and subject group for 180°/s could have been less reliable. The evidence of difference was also only moderately significant with a *p* value close to 0.05 (*p* = 0.042). Although a greater level of significance may be seen with an increased sample size for analysis [11], previously reported that even with a small sample size of 10, significant differences in PRD were observed at the rotation speed of 100°/s [11]. Therefore, sample size may not have been the limiting factor here.

From the results, preliminary evidences do show that PRD values were significantly different between control and subject groups. 180°/s Per-rotatory PRD is most significantly different (*p* = 0.005) followed by 50°/s post-rotatory PRD (CCW, *p* = 0.007; CW, *p* = 0.021) and log of 180°/s post-rotatory PRD (*p* = 0.042). No SPEV TC variables regardless of the speed of rotation are significantly different between control and subject group. There also seems to be an effect on speed of rotation on PRD as significant differences in the per-rotatory PRD between control and subject group, were only seen at 180°/s and not at 50°/s rotation. However, regression analysis only showed that CCW post-rotatory PRD at 50°/s was a strong predictor of MSS, holding all other independent variables constant.

## 5. Conclusions

SPEV TC in general was not observed to be significantly different between the control and subject group. The rotation speed (50°/s vs. 180°/s) also did not seem to influence the SPEV TC of both per and post rotation. Therefore, SPEV TC was not elevated in MSS individuals as hypothesized. However, since both SPEV TC and PRD had never been concurrently recorded, the interaction effects between both parameters were not accounted for. With simultaneous recording of both parameters in this study, PRD was observed to be significantly different between controls and subjects for both speeds of rotation. This supported the hypothesis that MSS individuals may have an elevated PRD as compared to controls. Although there were significant differences between groups for PRD, PRD was only a strong predictor of MSS at 50°/s and not at 180°/s after regression analysis. SPEV TC on the other hand, was seen to be a negligible predictor of MSS.

The prediction of MSS using regression analysis seemed to be consistent for SPEV TC, since between groups, no significant difference was noted. In conclusion, from the evaluation of SPEV TC, the reflexive eye movement did not seem to correlate well with MSS and thus, may not be a useful clinical indicator of MSS. The PRD on the other hand could be a possible clinical indicator of MSS as there was significant difference between controls and subjects. However, its prediction of MSS in general remains inconclusive due to the limitation of a small sample size. A small sample size faces the problem of poor external validity, which refers to the process of generalisation to make predictions about the entire population. Although, power calculation resulted in an acceptable sample size of 50 in this study, there may still be poor external validity because one or more of the groups for comparison have a sample size of less than 30. In addition, because of the small sample size, we were unable to include a more comprehensive age distribution to include the younger and older age groups. Although the effect of age on motion sickness susceptibility is controversial, it has been suggested that children less than 6 years of age have immature vestibular system and are hence immune to motion sickness, while older adults are less susceptible to motion sickness. A further analysis with a large sample size with age stratification may be able to provide more power in determining the strength of the relationship between CVS and MSS. Further studies with a larger sample size, may help to improve the PRD’s predictability of MSS and justify the strong evidence of difference observed in PRD between the control and subject group. However, it can only be said in this study, that there were some preliminary evidence to suggest a relationship between PRD and MSS.

Furthermore, the effects of the otolith in particular, the utricle and saccule are not known, as we did not perform the o-VEMP and did not analyse the results of the recorded c-VEMP. This is a limitation as the otoliths undeniable play a role in the production of motion sickness through its function of detecting linear acceleration and garvito-inertial changes. It would be interesting in future studies to evaluate the otoliths’ involvement with an Off-Vertical Axis Rotation (OVAR), in addition to analysing VEMP as greater otolith activities were reported in less MSS participants.

Since SPEV TC did not correlate with MSS, the use of SPEV TC to evaluate the efficacy of habituation training on MSS may yet be reliable. PRD on the other hand, could be a more reliable measure of habituation training efficacy and should be concurrently recorded with SPEV TC in future studies on habituation and motion sickness susceptibility.

## Figures and Tables

**Table 1:** *p* values, 95% confidence intervals with mean difference and post-hoc power for 50°/s.

Independent Variables	*p*-Values (3 dp)	95% Confidence Interval (2 dp)	Mean Difference in SECONDS [Control-Subject; 2 dp]	Post-hoc Observed Power
50°/s CW Per-rotatory SPEV TC	0.012	(0.73, 5.46)	3.10	0.986
50°/s CCW Per-rotatory SPEV TC	0.516	(−1.45, 2.82)	0.69	0.414
50°/s Averaged Post-rotatory SPEV TC	0.990	(−2.33, 2.36)	0.01	0.032
50°/s Averaged Per-rotatory PRD	0.502	(−10.35, 5.20)	−2.58	0.441
50°/s CW Post-rotatory PRD	0.021	(−18.10, −1.59)	−9.84	0.786
50°/s CCW Post-rotatory PRD	0.007	(−22.56, −3.96)	−13.56	0.784

CW: Clock-Wise, CCW: Counter Clock-Wise, SPEV: Slow-Phase Eye Velocity, TC; Time constant, PRD: Perceived Rotational Duration.

**Table 2:** *p* values, 95% confidence intervals with mean difference and post-hoc power for 180°/s.

Independent Variables	*p*-Values (3 dp)	95% Confidence Interval (2 dp)	Mean Difference in Seconds [Control-Subject; 2 dp]	Post-hoc Observed Power
180°/s CW Per-rotatory SPEV TC	0.248	(−0.79, 2.96)	1.08	0.339
180°/s CCW Per-rotatory SPEV TC	0.730	(−1.87, 1.32)	−0.27	0.066
180°/s Averaged 2^nd^ Recorded Per-rotatory SPEV TC	0.760	(−1.27, 1.72)	0.23	0.061
180°/s Averaged Post-rotatory SPEV TC	0.862	(−1.38, 1.64)	0.13	0.042
180°/s Averaged Per-rotatory PRD	0.005	(−17.86, −3.64)	−10.75	0.837
180°/s Log of Averaged Post-rotatory PRD	**0.042**	(−21.87, −0.45)	−11.16	0.799

CW: Clock-Wise, CCW: Counter Clock-Wise, SPEV: Slow-Phase Eye Velocity, TC; Time constant, PRD: Perceived Rotational Duration.

**Table 3:** Regression analysis *p* values, 95% confidence intervals with mean difference and strength for 50°/s.

Other Variables	*p* Values, 95% CI (2 dp)	Mean Difference (1 dp)	Strength of Predictor
Gender	0.067 (−1.08, 30.95)	14.9	Weak
Total Averaged Per-rotatory PRD	0.425 (−0.61, 1,42)	0.4	Poor
Log Total Averaged Post-rotatory PRD	0.772 (−59.69, 79.68)	10.0	Poor

PRD: Perceived Rotational Duration.

**Table 4:** Regression for 180°/s *p* values, 95% confidence intervals with mean difference and prediction strength.

Other Variables	*p* Values, 95% CI (2 dp)	Mean Difference (1 dp)	Strength of Predictor
Gender	0.056 (−0.61, 48.07)	23.7	Weak
Averaged Per-rotatory PRD	0.135 (−2.07, 0.30)	−0.9	Poor

PRD: Perceived Rotational Duration.
